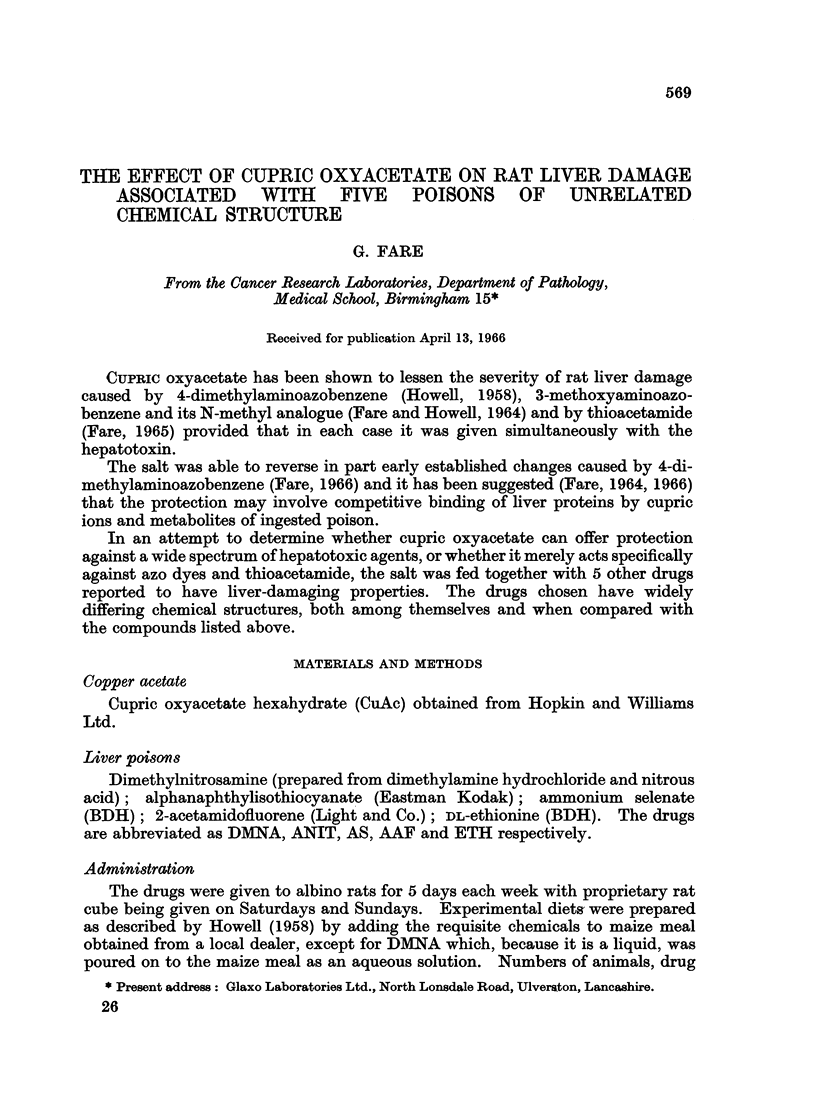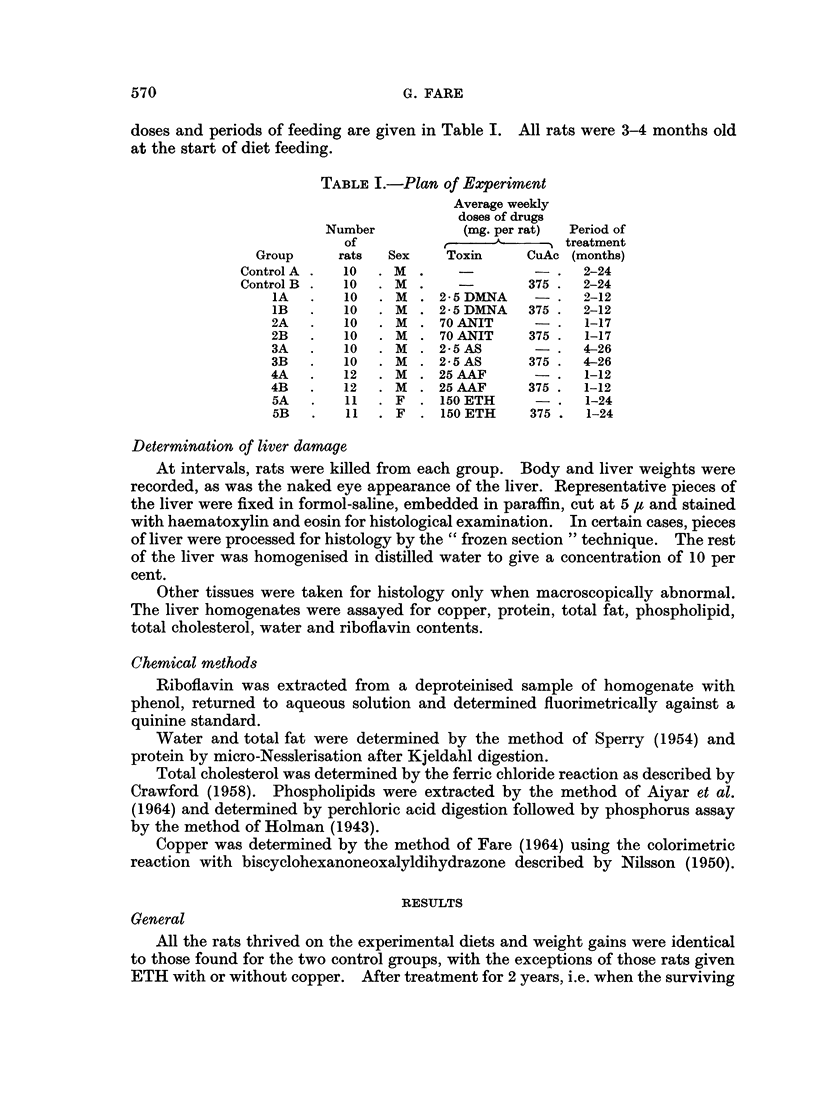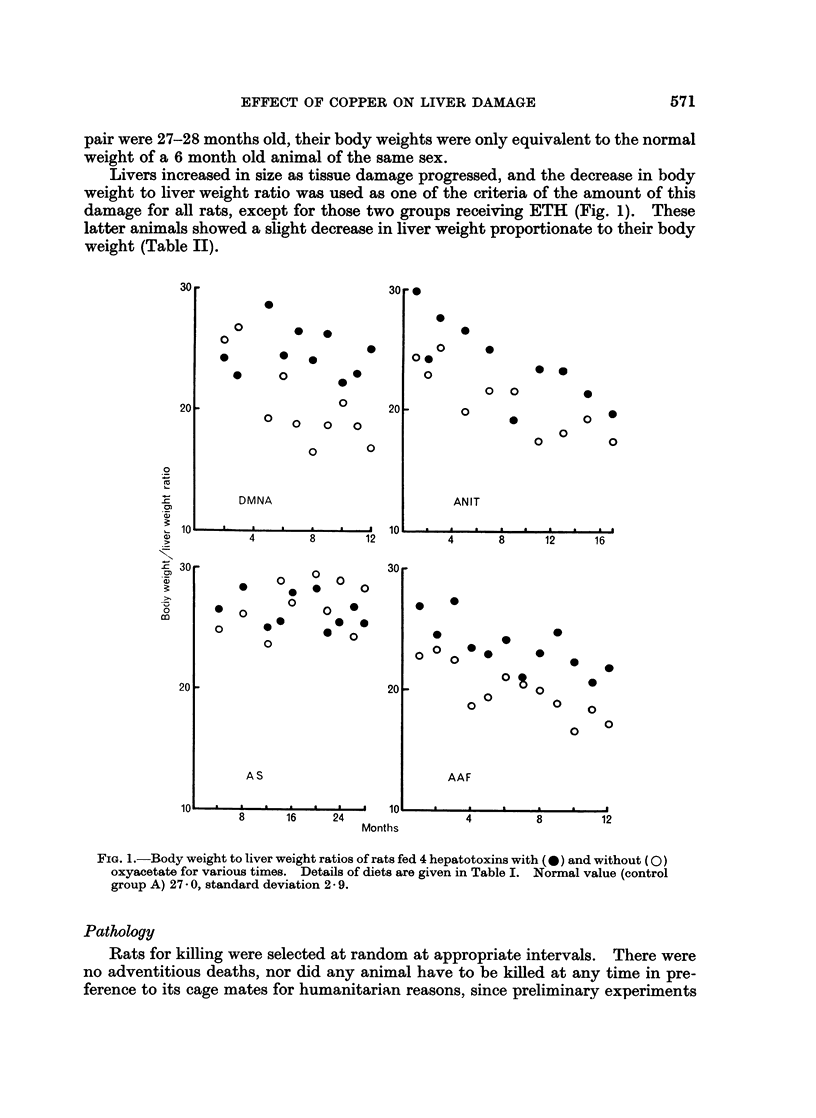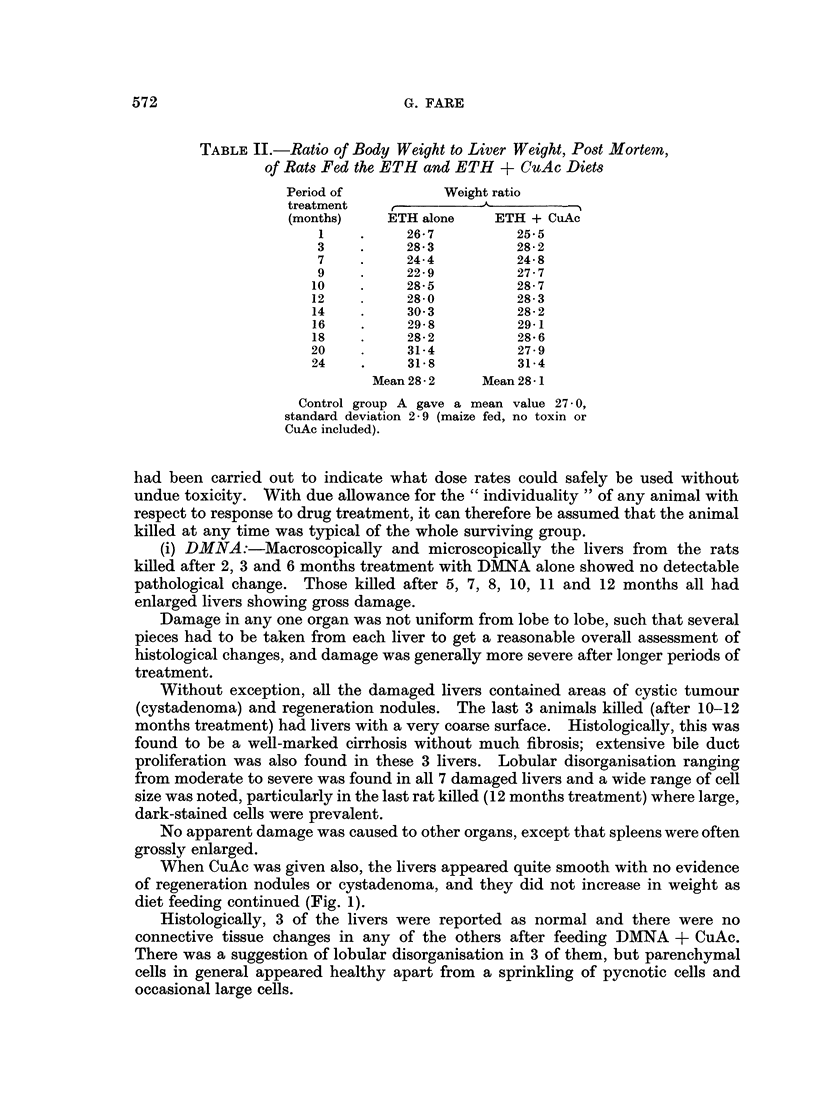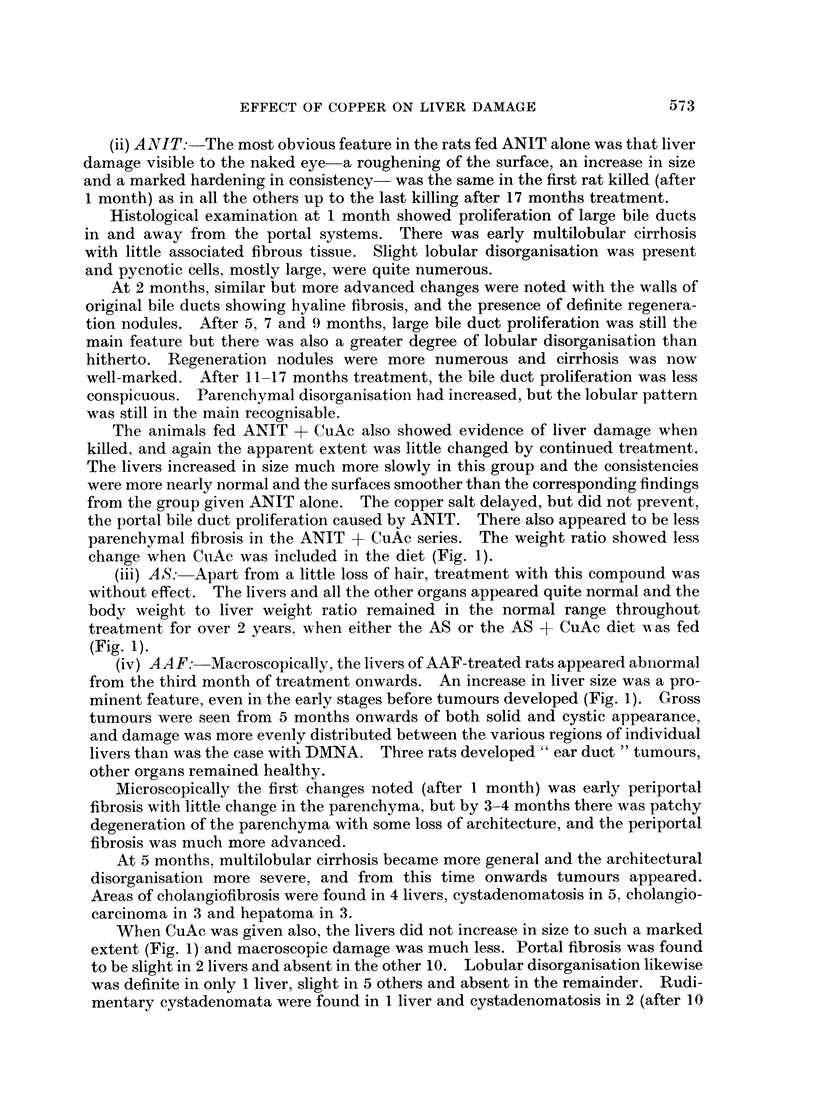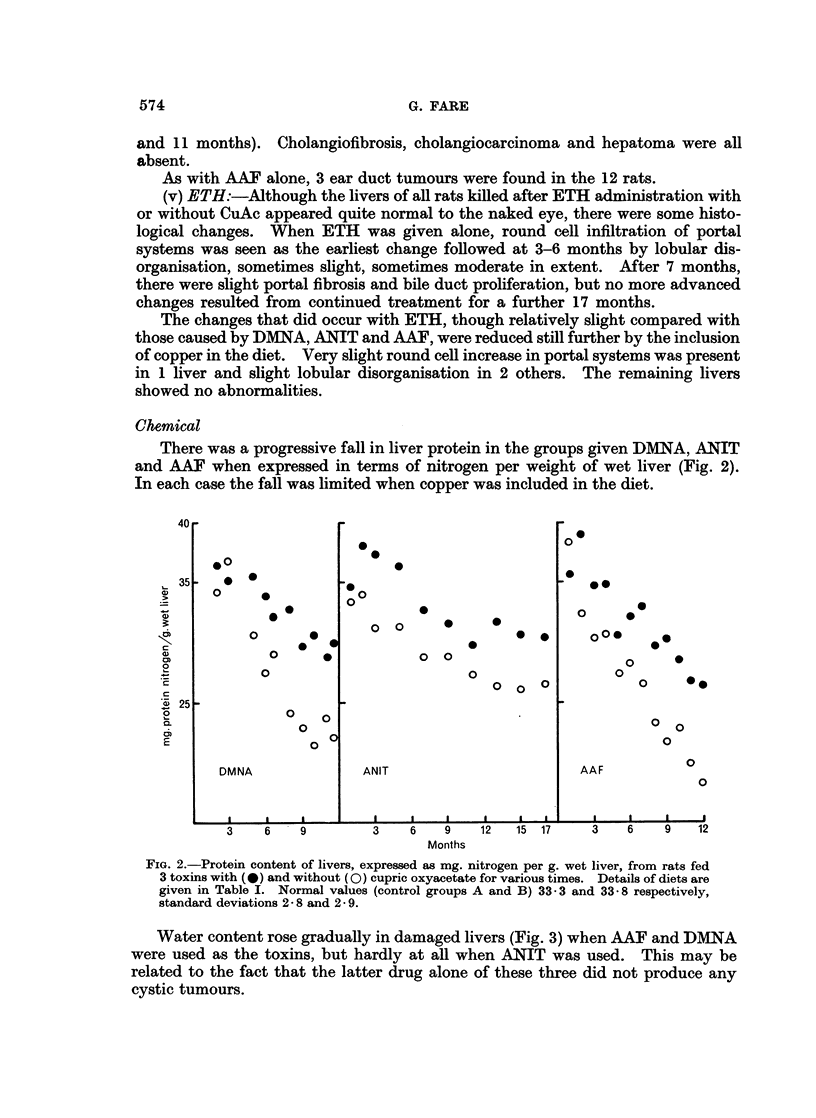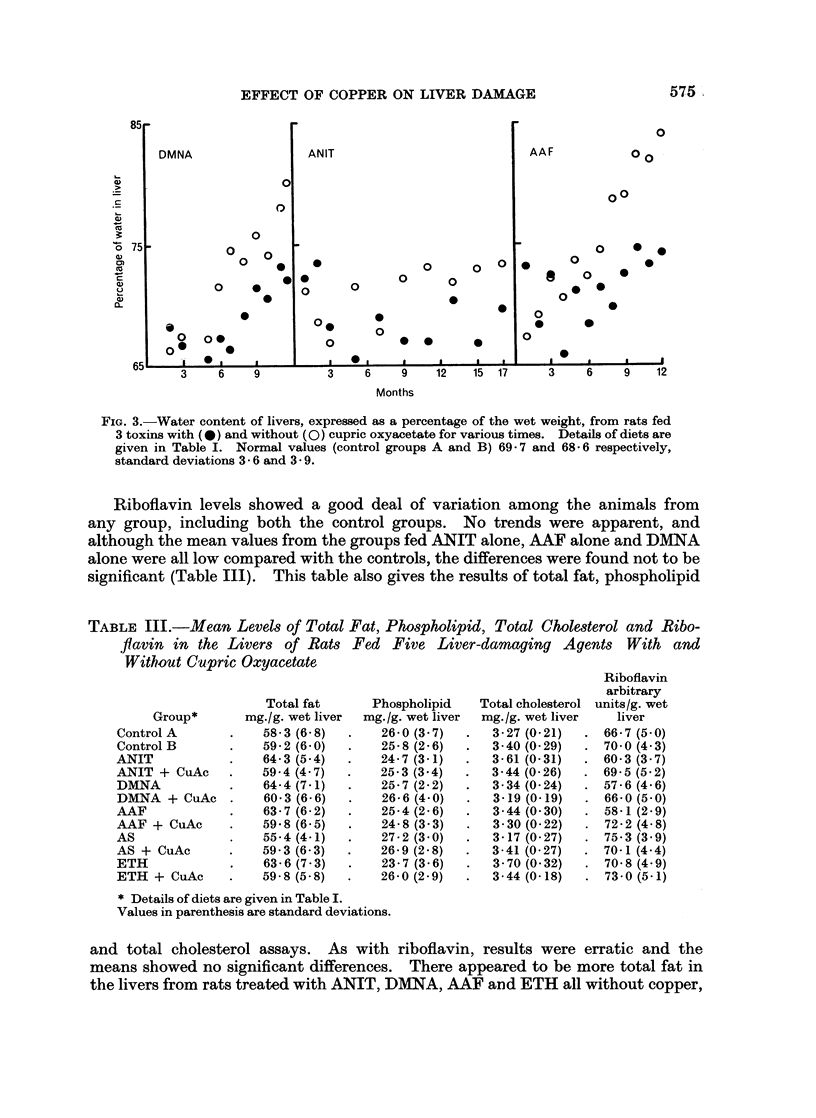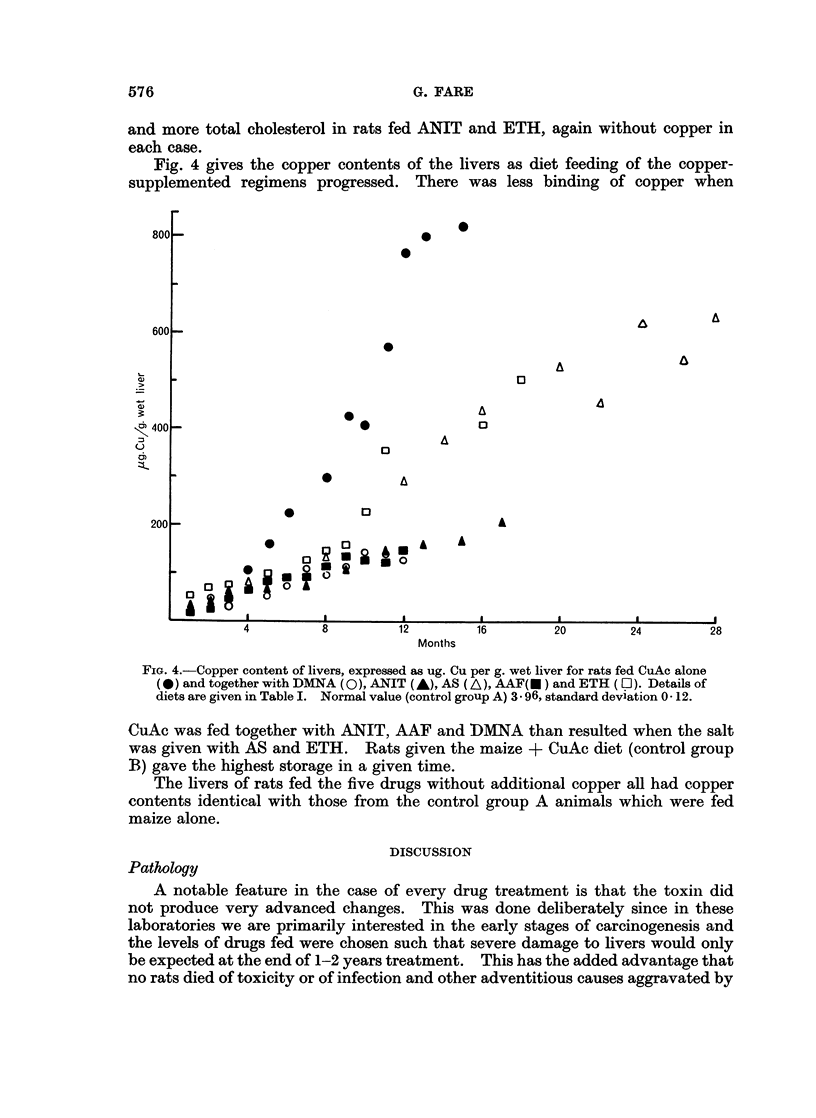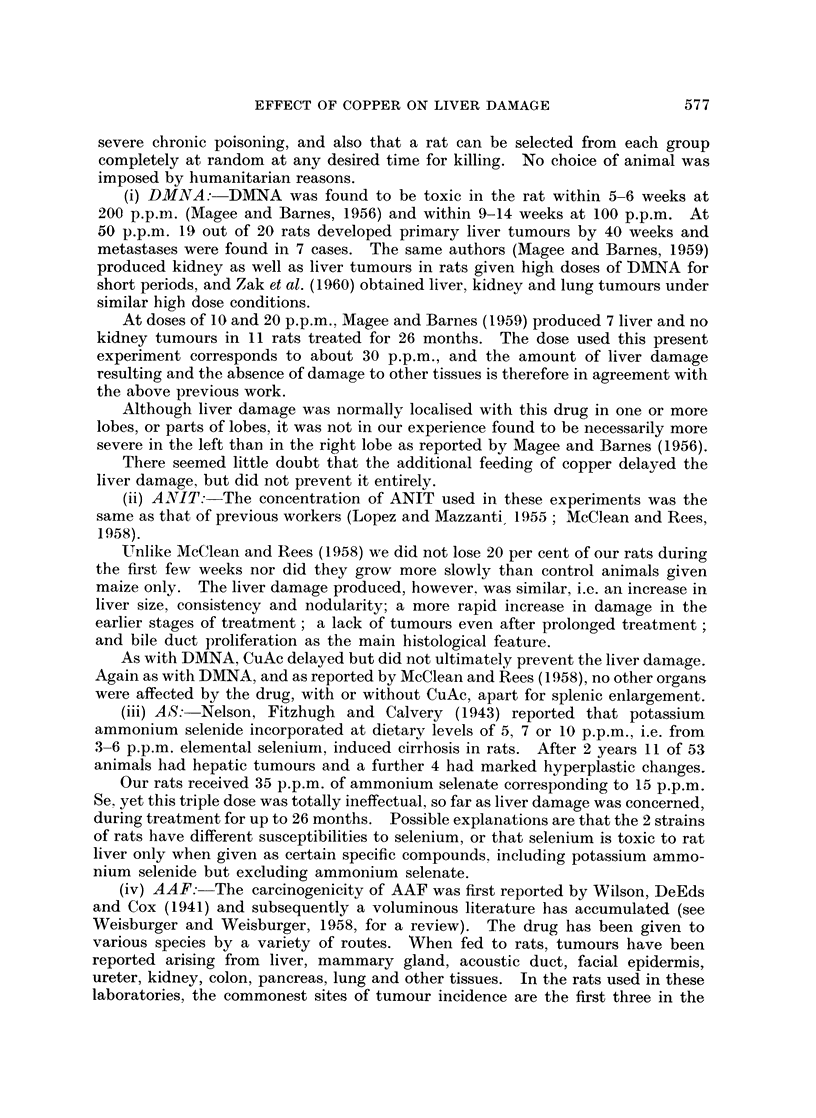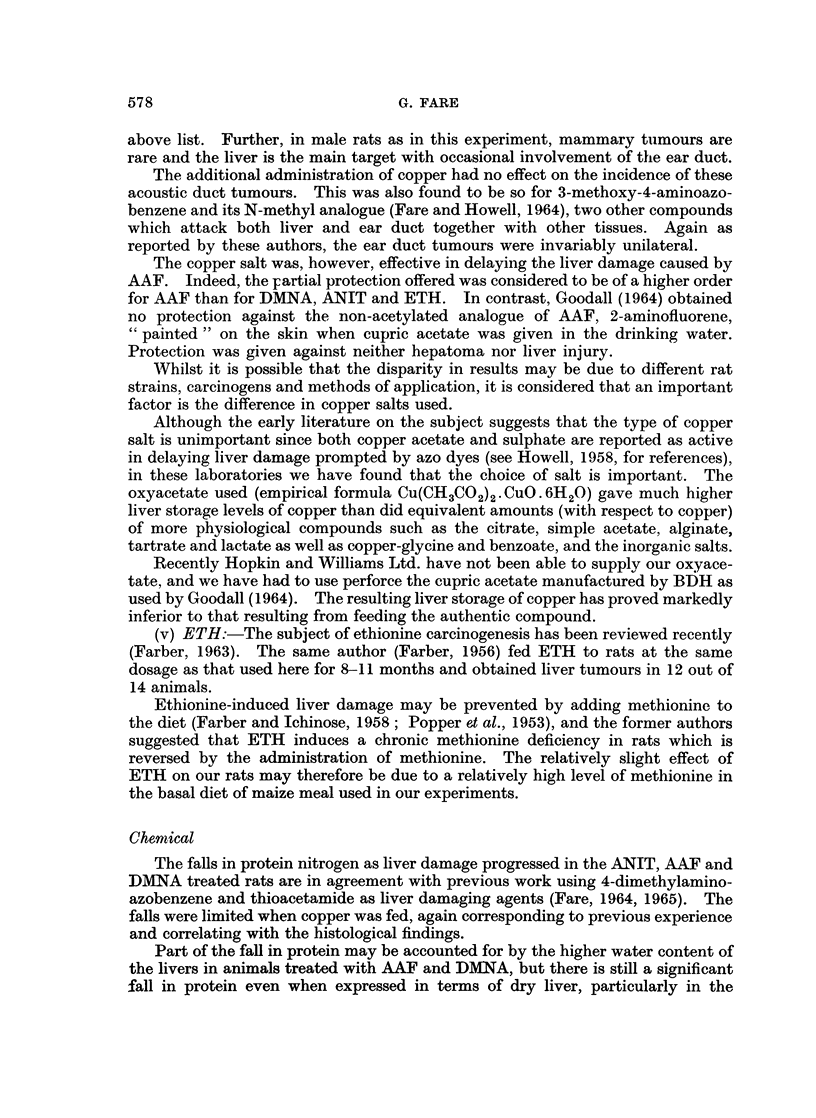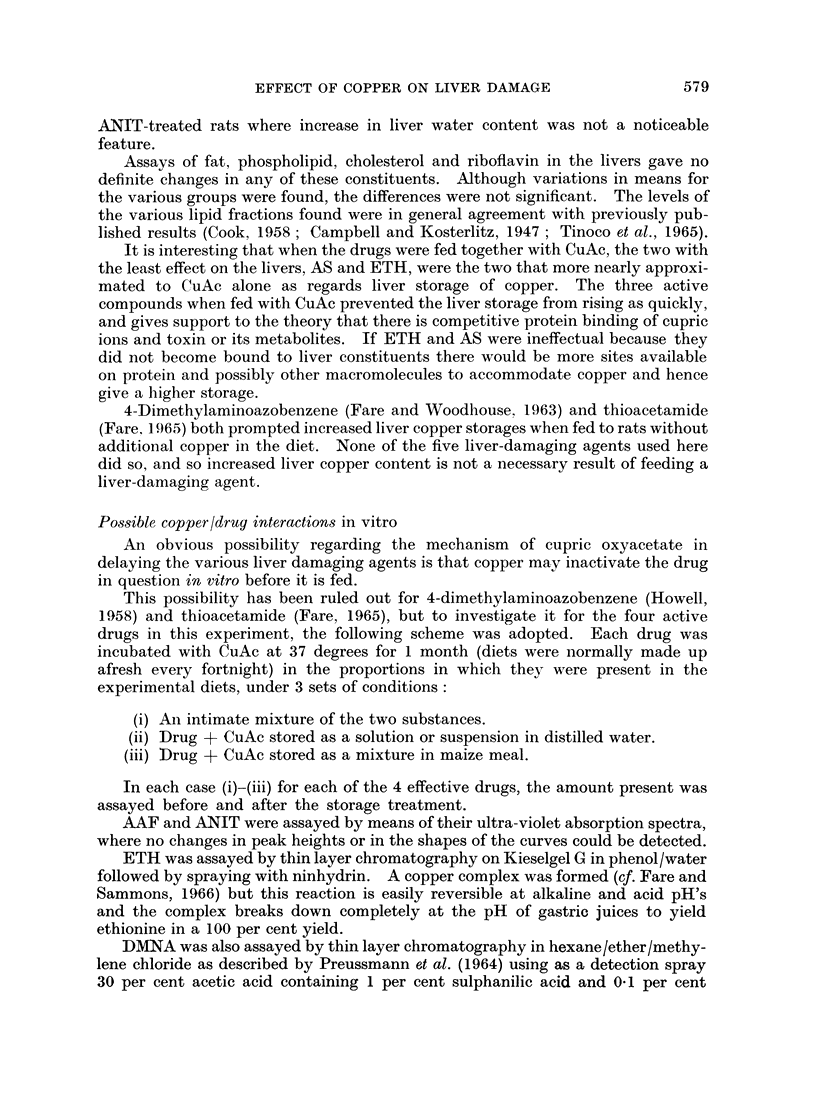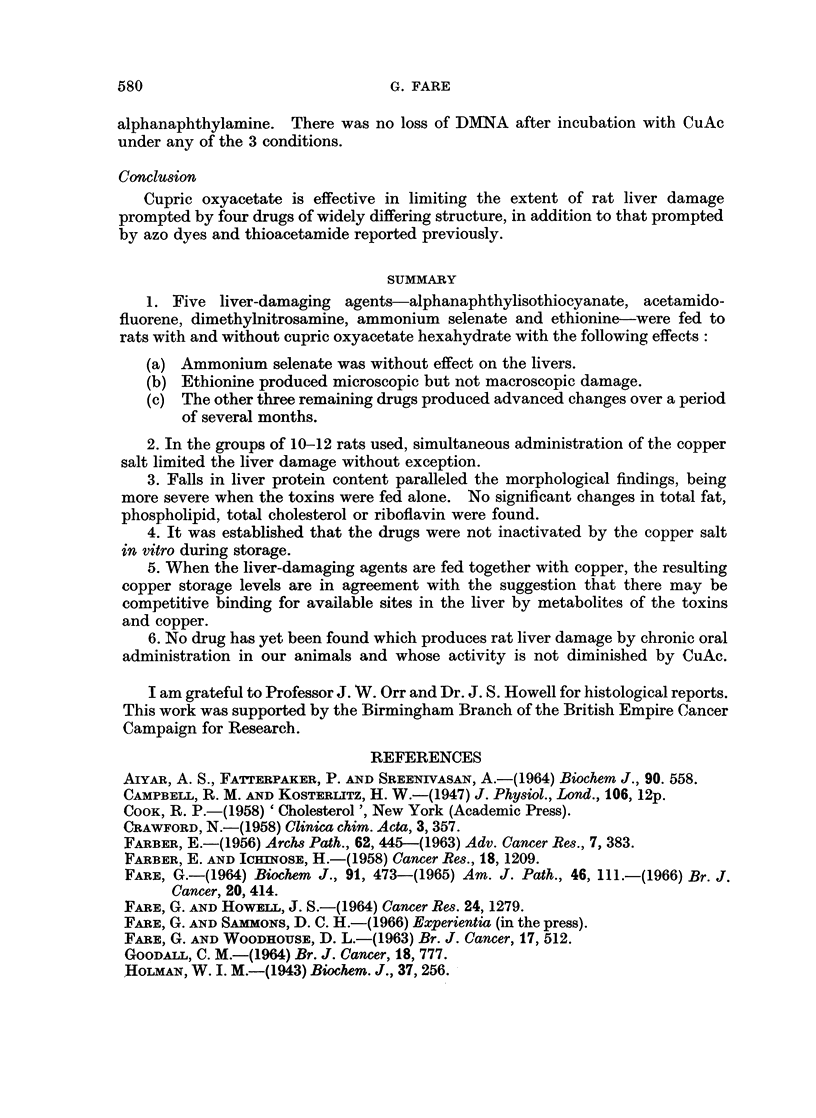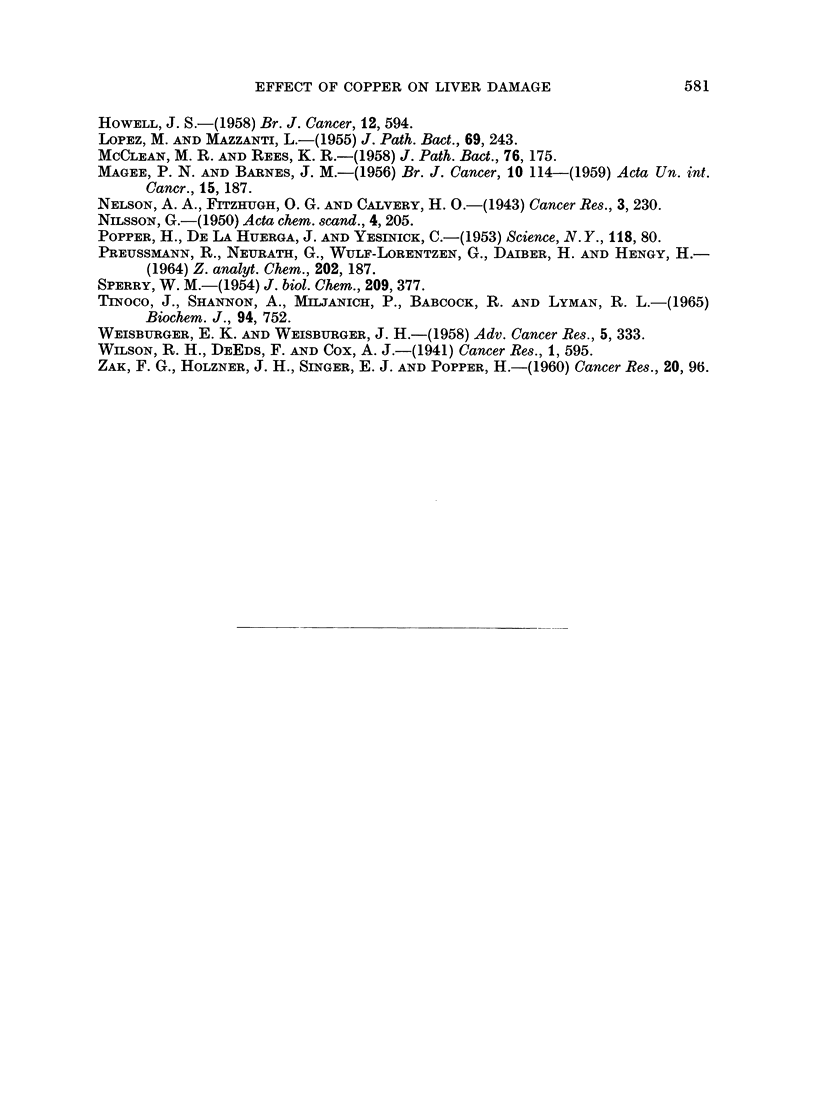# The effect of cupric oxyacetate on rat liver damage associated with five poisons of unrelated chemical structure.

**DOI:** 10.1038/bjc.1966.71

**Published:** 1966-09

**Authors:** G. Fare


### THE EFFECT OF CUPRIC OXYACETATE ON RAT LIVER DAMAGE ASSOCIATED WITH FIVE POISONS OF UNRELATED CHEMICAL STRUCTURE

G. FARE

*From the Cancer Research Laboratories, Department of Pathology, Medical School, Birmingham* 15*

Received for publication April 13, 1966

CUPRIC oxyacetate has been shown to lessen the severity of rat liver damage caused by 4-dimethylaminoazobenzene (Howell, 1958), 3-methoxyaminoazobenzene and its N-methyl analogue (Fare and Howell, 1964) and by thioacetamide (Fare, 1965) provided that in each case it was given simultaneously with the hepatotoxin.

The salt was able to reverse in part early established changes caused by 4-dimethylaminoazobenzene (Fare, 1966) and it has been suggested (Fare, 1964, 1966) that the protection may involve competitive binding of liver proteins by cupric ions and metabolites of ingested poison.

In an attempt to determine whether cupric oxyacetate can offer protection against a wide spectrum of hepatotoxic agents, or whether it merely acts specifically against azo dyes and thioacetamide, the salt was fed together with 5 other drugs reported to have liver-damaging properties. The drugs chosen have widely differing chemical structures, both among themselves and when compared with the compounds listed above.

### MATERIALS AND METHODS

### *Copper acetate*

Cupric oxyacetate hexahydrate (CuAc) obtained from Hopkin and Williams Ltd.

### *Liver poisons*

Dimethylnitrosamine (prepared from dimethylamine hydrochloride and nitrous acid); alphanaphthylisothiocyanate (Eastman Kodak); ammonium selenate (BDH); 2-acetamidofluorene (Light and Co.); DL-ethionine (BDH). The drugs are abbreviated as DMNA, ANIT, AS, AAF and ETH respectively.

### *Administration*

The drugs were given to albino rats for 5 days each week with proprietary rat cube being given on Saturdays and Sundays. Experimental diets were prepared as described by Howell (1958) by adding the requisite chemicals to maize meal obtained from a local dealer, except for DMNA which, because it is a liquid, was poured on to the maize meal as an aqueous solution. Numbers of animals, drug

* Present address: Glaxo Laboratories Ltd., North Lonsdale Road, Ulverston, Lancashire.

doses and periods of feeding are given in Table I. All rats were 3–4 months old at the start of diet feeding.

TABLE I.—*Plan of Experiment*

| Group | Number of rats | Sex | Average weekly doses of drugs (mg. per rat) Toxin | CuAc | Period of treatment (months) |
|---|---|---|---|---|---|
| Control A | 10 | M | — | — | 2–24 |
| Control B | 10 | M | — | 375 | 2–24 |
| 1A | 10 | M | 2·5 DMNA | — | 2–12 |
| 1B | 10 | M | 2·5 DMNA | 375 | 2–12 |
| 2A | 10 | M | 70 ANIT | — | 1–17 |
| 2B | 10 | M | 70 ANIT | 375 | 1–17 |
| 3A | 10 | M | 2·5 AS | — | 4–26 |
| 3B | 10 | M | 2·5 AS | 375 | 4–26 |
| 4A | 12 | M | 25 AAF | — | 1–12 |
| 4B | 12 | M | 25 AAF | 375 | 1–12 |
| 5A | 11 | F | 150 ETH | — | 1–24 |
| 5B | 11 | F | 150 ETH | 375 | 1–24 |

*Determination of liver damage*

At intervals, rats were killed from each group. Body and liver weights were recorded, as was the naked eye appearance of the liver. Representative pieces of the liver were fixed in formol-saline, embedded in paraffin, cut at 5 $\mu$ and stained with haematoxylin and eosin for histological examination. In certain cases, pieces of liver were processed for histology by the " frozen section " technique. The rest of the liver was homogenised in distilled water to give a concentration of 10 per cent.

Other tissues were taken for histology only when macroscopically abnormal. The liver homogenates were assayed for copper, protein, total fat, phospholipid, total cholesterol, water and riboflavin contents.

*Chemical methods*

Riboflavin was extracted from a deproteinised sample of homogenate with phenol, returned to aqueous solution and determined fluorimetrically against a quinine standard.

Water and total fat were determined by the method of Sperry (1954) and protein by micro-Nesslerisation after Kjeldahl digestion.

Total cholesterol was determined by the ferric chloride reaction as described by Crawford (1958). Phospholipids were extracted by the method of Aiyar *et al.* (1964) and determined by perchloric acid digestion followed by phosphorus assay by the method of Holman (1943).

Copper was determined by the method of Fare (1964) using the colorimetric reaction with biscyclohexanoneoxalyldihydrazone described by Nilsson (1950).

### RESULTS

*General*

All the rats thrived on the experimental diets and weight gains were identical to those found for the two control groups, with the exceptions of those rats given ETH with or without copper. After treatment for 2 years, i.e. when the surviving pair were 27–28 months old, their body weights were only equivalent to the normal weight of a 6 month old animal of the same sex.

Livers increased in size as tissue damage progressed, and the decrease in body weight to liver weight ratio was used as one of the criteria of the amount of this damage for all rats, except for those two groups receiving ETH (Fig. 1). These latter animals showed a slight decrease in liver weight proportionate to their body weight (Table II).

Fig. 1.—Body weight to liver weight ratios of rats fed 4 hepatotoxins with (●) and without (○) oxyacetate for various times. Details of diets are given in Table I. Normal value (control group A) 27·0, standard deviation 2·9.

*Pathology*

Rats for killing were selected at random at appropriate intervals. There were no adventitious deaths, nor did any animal have to be killed at any time in preference to its cage mates for humanitarian reasons, since preliminary experiments

TABLE II.—*Ratio of Body Weight to Liver Weight, Post Mortem, of Rats Fed the ETH and ETH + CuAc Diets*

| Period of treatment (months) | Weight ratio | |
|---|---|---|
| | ETH alone | ETH + CuAc |
| 1 | 26·7 | 25·5 |
| 3 | 28·3 | 28·2 |
| 7 | 24·4 | 24·8 |
| 9 | 22·9 | 27·7 |
| 10 | 28·5 | 28·7 |
| 12 | 28·0 | 28·3 |
| 14 | 30·3 | 28·2 |
| 16 | 29·8 | 29·1 |
| 18 | 28·2 | 28·6 |
| 20 | 31·4 | 27·9 |
| 24 | 31·8 | 31·4 |
| | Mean 28·2 | Mean 28·1 |

Control group A gave a mean value 27·0, standard deviation 2·9 (maize fed, no toxin or CuAc included).

had been carried out to indicate what dose rates could safely be used without undue toxicity. With due allowance for the " individuality " of any animal with respect to response to drug treatment, it can therefore be assumed that the animal killed at any time was typical of the whole surviving group.

(i) *DMNA*:—Macroscopically and microscopically the livers from the rats killed after 2, 3 and 6 months treatment with DMNA alone showed no detectable pathological change. Those killed after 5, 7, 8, 10, 11 and 12 months all had enlarged livers showing gross damage.

Damage in any one organ was not uniform from lobe to lobe, such that several pieces had to be taken from each liver to get a reasonable overall assessment of histological changes, and damage was generally more severe after longer periods of treatment.

Without exception, all the damaged livers contained areas of cystic tumour (cystadenoma) and regeneration nodules. The last 3 animals killed (after 10–12 months treatment) had livers with a very coarse surface. Histologically, this was found to be a well-marked cirrhosis without much fibrosis; extensive bile duct proliferation was also found in these 3 livers. Lobular disorganisation ranging from moderate to severe was found in all 7 damaged livers and a wide range of cell size was noted, particularly in the last rat killed (12 months treatment) where large, dark-stained cells were prevalent.

No apparent damage was caused to other organs, except that spleens were often grossly enlarged.

When CuAc was given also, the livers appeared quite smooth with no evidence of regeneration nodules or cystadenoma, and they did not increase in weight as diet feeding continued (Fig. 1).

Histologically, 3 of the livers were reported as normal and there were no connective tissue changes in any of the others after feeding DMNA + CuAc. There was a suggestion of lobular disorganisation in 3 of them, but parenchymal cells in general appeared healthy apart from a sprinkling of pycnotic cells and occasional large cells.

(ii) *ANIT:*—The most obvious feature in the rats fed ANIT alone was that liver damage visible to the naked eye—a roughening of the surface, an increase in size and a marked hardening in consistency— was the same in the first rat killed (after 1 month) as in all the others up to the last killing after 17 months treatment.

Histological examination at 1 month showed proliferation of large bile ducts in and away from the portal systems. There was early multilobular cirrhosis with little associated fibrous tissue. Slight lobular disorganisation was present and pycnotic cells, mostly large, were quite numerous.

At 2 months, similar but more advanced changes were noted with the walls of original bile ducts showing hyaline fibrosis, and the presence of definite regeneration nodules. After 5, 7 and 9 months, large bile duct proliferation was still the main feature but there was also a greater degree of lobular disorganisation than hitherto. Regeneration nodules were more numerous and cirrhosis was now well-marked. After 11–17 months treatment, the bile duct proliferation was less conspicuous. Parenchymal disorganisation had increased, but the lobular pattern was still in the main recognisable.

The animals fed ANIT + CuAc also showed evidence of liver damage when killed, and again the apparent extent was little changed by continued treatment. The livers increased in size much more slowly in this group and the consistencies were more nearly normal and the surfaces smoother than the corresponding findings from the group given ANIT alone. The copper salt delayed, but did not prevent, the portal bile duct proliferation caused by ANIT. There also appeared to be less parenchymal fibrosis in the ANIT + CuAc series. The weight ratio showed less change when CuAc was included in the diet (Fig. 1).

(iii) *AS:*—Apart from a little loss of hair, treatment with this compound was without effect. The livers and all the other organs appeared quite normal and the body weight to liver weight ratio remained in the normal range throughout treatment for over 2 years, when either the AS or the AS + CuAc diet was fed (Fig. 1).

(iv) *AAF:*—Macroscopically, the livers of AAF-treated rats appeared abnormal from the third month of treatment onwards. An increase in liver size was a prominent feature, even in the early stages before tumours developed (Fig. 1). Gross tumours were seen from 5 months onwards of both solid and cystic appearance, and damage was more evenly distributed between the various regions of individual livers than was the case with DMNA. Three rats developed " ear duct " tumours, other organs remained healthy.

Microscopically the first changes noted (after 1 month) was early periportal fibrosis with little change in the parenchyma, but by 3–4 months there was patchy degeneration of the parenchyma with some loss of architecture, and the periportal fibrosis was much more advanced.

At 5 months, multilobular cirrhosis became more general and the architectural disorganisation more severe, and from this time onwards tumours appeared. Areas of cholangiofibrosis were found in 4 livers, cystadenomatosis in 5, cholangiocarcinoma in 3 and hepatoma in 3.

When CuAc was given also, the livers did not increase in size to such a marked extent (Fig. 1) and macroscopic damage was much less. Portal fibrosis was found to be slight in 2 livers and absent in the other 10. Lobular disorganisation likewise was definite in only 1 liver, slight in 5 others and absent in the remainder. Rudimentary cystadenomata were found in 1 liver and cystadenomatosis in 2 (after 10

and 11 months). Cholangiofibrosis, cholangiocarcinoma and hepatoma were all absent.

As with AAF alone, 3 ear duct tumours were found in the 12 rats.

(v) *ETH:*—Although the livers of all rats killed after ETH administration with or without CuAc appeared quite normal to the naked eye, there were some histological changes. When ETH was given alone, round cell infiltration of portal systems was seen as the earliest change followed at 3–6 months by lobular disorganisation, sometimes slight, sometimes moderate in extent. After 7 months, there were slight portal fibrosis and bile duct proliferation, but no more advanced changes resulted from continued treatment for a further 17 months.

The changes that did occur with ETH, though relatively slight compared with those caused by DMNA, ANIT and AAF, were reduced still further by the inclusion of copper in the diet. Very slight round cell increase in portal systems was present in 1 liver and slight lobular disorganisation in 2 others. The remaining livers showed no abnormalities.

*Chemical*

There was a progressive fall in liver protein in the groups given DMNA, ANIT and AAF when expressed in terms of nitrogen per weight of wet liver (Fig. 2). In each case the fall was limited when copper was included in the diet.

FIG. 2.—Protein content of livers, expressed as mg. nitrogen per g. wet liver, from rats fed 3 toxins with (●) and without (○) cupric oxyacetate for various times. Details of diets are given in Table I. Normal values (control groups A and B) 33·3 and 33·8 respectively, standard deviations 2·8 and 2·9.

Water content rose gradually in damaged livers (Fig. 3) when AAF and DMNA were used as the toxins, but hardly at all when ANIT was used. This may be related to the fact that the latter drug alone of these three did not produce any cystic tumours.

FIG. 3.—Water content of livers, expressed as a percentage of the wet weight, from rats fed 3 toxins with (●) and without (○) cupric oxyacetate for various times. Details of diets are given in Table I. Normal values (control groups A and B) 69·7 and 68·6 respectively, standard deviations 3·6 and 3·9.

Riboflavin levels showed a good deal of variation among the animals from any group, including both the control groups. No trends were apparent, and although the mean values from the groups fed ANIT alone, AAF alone and DMNA alone were all low compared with the controls, the differences were found not to be significant (Table III). This table also gives the results of total fat, phospholipid

TABLE III.—*Mean Levels of Total Fat, Phospholipid, Total Cholesterol and Riboflavin in the Livers of Rats Fed Five Liver-damaging Agents With and Without Cupric Oxyacetate*

| Group* | Total fat mg./g. wet liver | Phospholipid mg./g. wet liver | Total cholesterol mg./g. wet liver | Riboflavin arbitrary units/g. wet liver |
|---|---|---|---|---|
| Control A | 58·3 (6·8) | 26·0 (3·7) | 3·27 (0·21) | 66·7 (5·0) |
| Control B | 59·2 (6·0) | 25·8 (2·6) | 3·40 (0·29) | 70·0 (4·3) |
| ANIT | 64·3 (5·4) | 24·7 (3·1) | 3·61 (0·31) | 60·3 (3·7) |
| ANIT + CuAc | 59·4 (4·7) | 25·3 (3·4) | 3·44 (0·26) | 69·5 (5·2) |
| DMNA | 64·4 (7·1) | 25·7 (2·2) | 3·34 (0·24) | 57·6 (4·6) |
| DMNA + CuAc | 60·3 (6·6) | 26·6 (4·0) | 3·19 (0·19) | 66·0 (5·0) |
| AAF | 63·7 (6·2) | 25·4 (2·6) | 3·44 (0·30) | 58·1 (2·9) |
| AAF + CuAc | 59·8 (6·5) | 24·8 (3·3) | 3·30 (0·22) | 72·2 (4·8) |
| AS | 55·4 (4·1) | 27·2 (3·0) | 3·17 (0·27) | 75·3 (3·9) |
| AS + CuAc | 59·3 (6·3) | 26·9 (2·8) | 3·41 (0·27) | 70·1 (4·4) |
| ETH | 63·6 (7·3) | 23·7 (3·6) | 3·70 (0·32) | 70·8 (4·9) |
| ETH + CuAc | 59·8 (5·8) | 26·0 (2·9) | 3·44 (0·18) | 73·0 (5·1) |

* Details of diets are given in Table I.
Values in parenthesis are standard deviations.

and total cholesterol assays. As with riboflavin, results were erratic and the means showed no significant differences. There appeared to be more total fat in the livers from rats treated with ANIT, DMNA, AAF and ETH all without copper, and more total cholesterol in rats fed ANIT and ETH, again without copper in each case.

Fig. 4 gives the copper contents of the livers as diet feeding of the copper-supplemented regimens progressed. There was less binding of copper when

FIG. 4.—Copper content of livers, expressed as ug. Cu per g. wet liver for rats fed CuAc alone (●) and together with DMNA (○), ANIT (▲), AS (△), AAF(■) and ETH (□). Details of diets are given in Table I. Normal value (control group A) 3·96, standard deviation 0·12.

CuAc was fed together with ANIT, AAF and DMNA than resulted when the salt was given with AS and ETH. Rats given the maize + CuAc diet (control group B) gave the highest storage in a given time.

The livers of rats fed the five drugs without additional copper all had copper contents identical with those from the control group A animals which were fed maize alone.

### DISCUSSION

### *Pathology*

A notable feature in the case of every drug treatment is that the toxin did not produce very advanced changes. This was done deliberately since in these laboratories we are primarily interested in the early stages of carcinogenesis and the levels of drugs fed were chosen such that severe damage to livers would only be expected at the end of 1–2 years treatment. This has the added advantage that no rats died of toxicity or of infection and other adventitious causes aggravated by severe chronic poisoning, and also that a rat can be selected from each group completely at random at any desired time for killing. No choice of animal was imposed by humanitarian reasons.

(i) *DMNA:*—DMNA was found to be toxic in the rat within 5–6 weeks at 200 p.p.m. (Magee and Barnes, 1956) and within 9–14 weeks at 100 p.p.m. At 50 p.p.m. 19 out of 20 rats developed primary liver tumours by 40 weeks and metastases were found in 7 cases. The same authors (Magee and Barnes, 1959) produced kidney as well as liver tumours in rats given high doses of DMNA for short periods, and Zak *et al.* (1960) obtained liver, kidney and lung tumours under similar high dose conditions.

At doses of 10 and 20 p.p.m., Magee and Barnes (1959) produced 7 liver and no kidney tumours in 11 rats treated for 26 months. The dose used this present experiment corresponds to about 30 p.p.m., and the amount of liver damage resulting and the absence of damage to other tissues is therefore in agreement with the above previous work.

Although liver damage was normally localised with this drug in one or more lobes, or parts of lobes, it was not in our experience found to be necessarily more severe in the left than in the right lobe as reported by Magee and Barnes (1956).

There seemed little doubt that the additional feeding of copper delayed the liver damage, but did not prevent it entirely.

(ii) *ANIT:*—The concentration of ANIT used in these experiments was the same as that of previous workers (Lopez and Mazzanti, 1955 ; McClean and Rees, 1958).

Unlike McClean and Rees (1958) we did not lose 20 per cent of our rats during the first few weeks nor did they grow more slowly than control animals given maize only. The liver damage produced, however, was similar, i.e. an increase in liver size, consistency and nodularity; a more rapid increase in damage in the earlier stages of treatment ; a lack of tumours even after prolonged treatment ; and bile duct proliferation as the main histological feature.

As with DMNA, CuAc delayed but did not ultimately prevent the liver damage. Again as with DMNA, and as reported by McClean and Rees (1958), no other organs were affected by the drug, with or without CuAc, apart for splenic enlargement.

(iii) *AS:*—Nelson, Fitzhugh and Calvery (1943) reported that potassium ammonium selenide incorporated at dietary levels of 5, 7 or 10 p.p.m., i.e. from 3–6 p.p.m. elemental selenium, induced cirrhosis in rats. After 2 years 11 of 53 animals had hepatic tumours and a further 4 had marked hyperplastic changes.

Our rats received 35 p.p.m. of ammonium selenate corresponding to 15 p.p.m. Se, yet this triple dose was totally ineffectual, so far as liver damage was concerned, during treatment for up to 26 months. Possible explanations are that the 2 strains of rats have different susceptibilities to selenium, or that selenium is toxic to rat liver only when given as certain specific compounds, including potassium ammonium selenide but excluding ammonium selenate.

(iv) *AAF:*—The carcinogenicity of AAF was first reported by Wilson, DeEds and Cox (1941) and subsequently a voluminous literature has accumulated (see Weisburger and Weisburger, 1958, for a review). The drug has been given to various species by a variety of routes. When fed to rats, tumours have been reported arising from liver, mammary gland, acoustic duct, facial epidermis, ureter, kidney, colon, pancreas, lung and other tissues. In the rats used in these laboratories, the commonest sites of tumour incidence are the first three in the above list. Further, in male rats as in this experiment, mammary tumours are rare and the liver is the main target with occasional involvement of the ear duct.

The additional administration of copper had no effect on the incidence of these acoustic duct tumours. This was also found to be so for 3-methoxy-4-aminoazobenzene and its N-methyl analogue (Fare and Howell, 1964), two other compounds which attack both liver and ear duct together with other tissues. Again as reported by these authors, the ear duct tumours were invariably unilateral.

The copper salt was, however, effective in delaying the liver damage caused by AAF. Indeed, the partial protection offered was considered to be of a higher order for AAF than for DMNA, ANIT and ETH. In contrast, Goodall (1964) obtained no protection against the non-acetylated analogue of AAF, 2-aminofluorene, " painted " on the skin when cupric acetate was given in the drinking water. Protection was given against neither hepatoma nor liver injury.

Whilst it is possible that the disparity in results may be due to different rat strains, carcinogens and methods of application, it is considered that an important factor is the difference in copper salts used.

Although the early literature on the subject suggests that the type of copper salt is unimportant since both copper acetate and sulphate are reported as active in delaying liver damage prompted by azo dyes (see Howell, 1958, for references), in these laboratories we have found that the choice of salt is important. The oxyacetate used (empirical formula $Cu(CH_3CO_2)_2 . CuO . 6H_2O$) gave much higher liver storage levels of copper than did equivalent amounts (with respect to copper) of more physiological compounds such as the citrate, simple acetate, alginate, tartrate and lactate as well as copper-glycine and benzoate, and the inorganic salts.

Recently Hopkin and Williams Ltd. have not been able to supply our oxyacetate, and we have had to use perforce the cupric acetate manufactured by BDH as used by Goodall (1964). The resulting liver storage of copper has proved markedly inferior to that resulting from feeding the authentic compound.

(v) *ETH:*—The subject of ethionine carcinogenesis has been reviewed recently (Farber, 1963). The same author (Farber, 1956) fed ETH to rats at the same dosage as that used here for 8–11 months and obtained liver tumours in 12 out of 14 animals.

Ethionine-induced liver damage may be prevented by adding methionine to the diet (Farber and Ichinose, 1958 ; Popper *et al.*, 1953), and the former authors suggested that ETH induces a chronic methionine deficiency in rats which is reversed by the administration of methionine. The relatively slight effect of ETH on our rats may therefore be due to a relatively high level of methionine in the basal diet of maize meal used in our experiments.

*Chemical*

The falls in protein nitrogen as liver damage progressed in the ANIT, AAF and DMNA treated rats are in agreement with previous work using 4-dimethylaminoazobenzene and thioacetamide as liver damaging agents (Fare, 1964, 1965). The falls were limited when copper was fed, again corresponding to previous experience and correlating with the histological findings.

Part of the fall in protein may be accounted for by the higher water content of the livers in animals treated with AAF and DMNA, but there is still a significant fall in protein even when expressed in terms of dry liver, particularly in the

ANIT-treated rats where increase in liver water content was not a noticeable feature.

Assays of fat, phospholipid, cholesterol and riboflavin in the livers gave no definite changes in any of these constituents. Although variations in means for the various groups were found, the differences were not significant. The levels of the various lipid fractions found were in general agreement with previously published results (Cook, 1958 ; Campbell and Kosterlitz, 1947 ; Tinoco *et al.*, 1965).

It is interesting that when the drugs were fed together with CuAc, the two with the least effect on the livers, AS and ETH, were the two that more nearly approximated to CuAc alone as regards liver storage of copper. The three active compounds when fed with CuAc prevented the liver storage from rising as quickly, and gives support to the theory that there is competitive protein binding of cupric ions and toxin or its metabolites. If ETH and AS were ineffectual because they did not become bound to liver constituents there would be more sites available on protein and possibly other macromolecules to accommodate copper and hence give a higher storage.

4-Dimethylaminoazobenzene (Fare and Woodhouse, 1963) and thioacetamide (Fare, 1965) both prompted increased liver copper storages when fed to rats without additional copper in the diet. None of the five liver-damaging agents used here did so, and so increased liver copper content is not a necessary result of feeding a liver-damaging agent.

*Possible copper/drug interactions* in vitro

An obvious possibility regarding the mechanism of cupric oxyacetate in delaying the various liver damaging agents is that copper may inactivate the drug in question *in vitro* before it is fed.

This possibility has been ruled out for 4-dimethylaminoazobenzene (Howell, 1958) and thioacetamide (Fare, 1965), but to investigate it for the four active drugs in this experiment, the following scheme was adopted. Each drug was incubated with CuAc at 37 degrees for 1 month (diets were normally made up afresh every fortnight) in the proportions in which they were present in the experimental diets, under 3 sets of conditions :

(i) An intimate mixture of the two substances.
(ii) Drug + CuAc stored as a solution or suspension in distilled water.
(iii) Drug + CuAc stored as a mixture in maize meal.

In each case (i)–(iii) for each of the 4 effective drugs, the amount present was assayed before and after the storage treatment.

AAF and ANIT were assayed by means of their ultra-violet absorption spectra, where no changes in peak heights or in the shapes of the curves could be detected.

ETH was assayed by thin layer chromatography on Kieselgel G in phenol/water followed by spraying with ninhydrin. A copper complex was formed (*cf.* Fare and Sammons, 1966) but this reaction is easily reversible at alkaline and acid pH's and the complex breaks down completely at the pH of gastric juices to yield ethionine in a 100 per cent yield.

DMNA was also assayed by thin layer chromatography in hexane/ether/methylene chloride as described by Preussmann *et al.* (1964) using as a detection spray 30 per cent acetic acid containing 1 per cent sulphanilic acid and 0·1 per cent alphanaphthylamine. There was no loss of DMNA after incubation with CuAc under any of the 3 conditions.

*Conclusion*

Cupric oxyacetate is effective in limiting the extent of rat liver damage prompted by four drugs of widely differing structure, in addition to that prompted by azo dyes and thioacetamide reported previously.

### SUMMARY

1. Five liver-damaging agents—alphanaphthylisothiocyanate, acetamidofluorene, dimethylnitrosamine, ammonium selenate and ethionine—were fed to rats with and without cupric oxyacetate hexahydrate with the following effects :

(a) Ammonium selenate was without effect on the livers.
(b) Ethionine produced microscopic but not macroscopic damage.
(c) The other three remaining drugs produced advanced changes over a period of several months.

2. In the groups of 10–12 rats used, simultaneous administration of the copper salt limited the liver damage without exception.

3. Falls in liver protein content paralleled the morphological findings, being more severe when the toxins were fed alone. No significant changes in total fat, phospholipid, total cholesterol or riboflavin were found.

4. It was established that the drugs were not inactivated by the copper salt *in vitro* during storage.

5. When the liver-damaging agents are fed together with copper, the resulting copper storage levels are in agreement with the suggestion that there may be competitive binding for available sites in the liver by metabolites of the toxins and copper.

6. No drug has yet been found which produces rat liver damage by chronic oral administration in our animals and whose activity is not diminished by CuAc.

I am grateful to Professor J. W. Orr and Dr. J. S. Howell for histological reports. This work was supported by the Birmingham Branch of the British Empire Cancer Campaign for Research.

### REFERENCES

AIYAR, A. S., FATTERPAKER, P. AND SREENIVASAN, A.—(1964) *Biochem J.*, **90**. 558.
CAMPBELL, R. M. AND KOSTERLITZ, H. W.—(1947) *J. Physiol., Lond.*, **106**, 12p.
COOK, R. P.—(1958) ' Cholesterol ', New York (Academic Press).
CRAWFORD, N.—(1958) *Clinica chim. Acta*, **3**, 357.
FARBER, E.—(1956) *Archs Path.*, **62**, 445—(1963) *Adv. Cancer Res.*, **7**, 383.
FARBER, E. AND ICHINOSE, H.—(1958) *Cancer Res.*, **18**, 1209.
FARE, G.—(1964) *Biochem J.*, **91**, 473—(1965) *Am. J. Path.*, **46**, 111.—(1966) *Br. J. Cancer*, **20**, 414.
FARE, G. AND HOWELL, J. S.—(1964) *Cancer Res.* **24**, 1279.
FARE, G. AND SAMMONS, D. C. H.—(1966) *Experientia* (in the press).
FARE, G. AND WOODHOUSE, D. L.—(1963) *Br. J. Cancer*, **17**, 512.
GOODALL, C. M.—(1964) *Br. J. Cancer*, **18**, 777.
HOLMAN, W. I. M.—(1943) *Biochem. J.*, **37**, 256.

HOWELL, J. S.—(1958) *Br. J. Cancer*, **12**, 594.
LOPEZ, M. AND MAZZANTI, L.—(1955) *J. Path. Bact.*, **69**, 243.
McCLEAN, M. R. AND REES, K. R.—(1958) *J. Path. Bact.*, **76**, 175.
MAGEE, P. N. AND BARNES, J. M.—(1956) *Br. J. Cancer*, **10** 114—(1959) *Acta Un. int. Cancr.*, **15**, 187.
NELSON, A. A., FITZHUGH, O. G. AND CALVERY, H. O.—(1943) *Cancer Res.*, **3**, 230.
NILSSON, G.—(1950) *Acta chem. scand.*, **4**, 205.
POPPER, H., DE LA HUERGA, J. AND YESINICK, C.—(1953) *Science, N.Y.*, **118**, 80.
PREUSSMANN, R., NEURATH, G., WULF-LORENTZEN, G., DAIBER, H. AND HENGY, H.—(1964) *Z. analyt. Chem.*, **202**, 187.
SPERRY, W. M.—(1954) *J. biol. Chem.*, **209**, 377.
TINOCO, J., SHANNON, A., MILJANICH, P., BABCOCK, R. AND LYMAN, R. L.—(1965) *Biochem. J.*, **94**, 752.
WEISBURGER, E. K. AND WEISBURGER, J. H.—(1958) *Adv. Cancer Res.*, **5**, 333.
WILSON, R. H., DEEDS, F. AND COX, A. J.—(1941) *Cancer Res.*, **1**, 595.
ZAK, F. G., HOLZNER, J. H., SINGER, E. J. AND POPPER, H.—(1960) *Cancer Res.*, **20**, 96.